# A Real-World Experience of Cemiplimab in Patients with Advanced Cutaneous Squamous Cell Carcinoma

**DOI:** 10.3390/cancers18030454

**Published:** 2026-01-30

**Authors:** Matteo Ravara, Tommaso Sani, Vincenzo D’Alonzo, Monica Valente, Elisa Cinotti, Clelia Miracco, Maura Colucci, Valentina Croce, Eleonora Carbonari, Ramiz Rana, Olindo Massarelli, Giovanni Rubino, Diana Giannarelli, Roberto Cuomo, Luca Grimaldi, Pietro Rubegni, Michele Maio, Anna Maria Di Giacomo

**Affiliations:** 1 Department of Medicine, Surgery and Neurosciences, University of Siena, 53100 Siena, Italy; m.ravara@student.unisi.it (M.R.); tommaso.sani@student.unisi.it (T.S.); vincenzo.dalonzo@student.unisi.it (V.D.); maura.colucci@student.unisi.it (M.C.); v.croce@student.unisi.it (V.C.); eleonora.carbonar@student.unisi.it (E.C.); r.rana1@student.unisi.it (R.R.); maio@unisi.it (M.M.); 2 Center for Immuno-Oncology, University Hospital of Siena, 53100 Siena, Italy; monica.valente@ao-siena.toscana.it; 3 Dermatology Unit, Department of Medicine, Surgery and Neuroscience, University Hospital of Siena, 53100 Siena, Italy; elisa.cinotti@unisi.it (E.C.); pietro.rubegni@unisi.it (P.R.); 4 Department of Pathology, University Hospital of Siena, 53100 Siena, Italy; clelia.miracco@unisi.it; 5 Maxillofacial Surgery Unit, Department of Medical Biotechnology, University Hospital of Siena, 53100 Siena, Italy; olindo.massarelli@ao-siena.toscana.it; 6 Unit of Radiation Oncology, University Hospital of Siena, 53100 Siena, Italy; g.rubino@ao-siena.toscana.it; 7 Department of Statistics, Fondazione Policlinico Universitario A. Gemelli IRCCS, 00168 Rome, Italy; diana.giannarelli@unicatt.it; 8 Plastic Surgery Unit, Department of Medicine, Surgery and Neuroscience, University Hospital of Siena, 53100 Siena, Italy; roberto.cuomo2@unisi.it (R.C.); luca.grimaldi@unisi.it (L.G.)

**Keywords:** immunotherapy, cutaneous squamous cell carcinoma, real-world experience, cemiplimab, skin cancer, elderly patients

## Abstract

Background: Cutaneous squamous cell carcinoma (cSCC) represents the second most common form of non-melanoma skin malignancy, and, when not amenable to curative surgery or radiotherapy, it is a life-threatening disease. The anti-PD-1 monoclonal antibody cemiplimab has transformed the outcome of advanced or metastatic cSCC, with response rates approaching 50% and sustained benefit beyond three years in clinical trials. Cemiplimab is now the first-line standard of care treatment for advanced disease. Methods: This retrospective observational study included consecutive adult patients with locally advanced (lac) or metastatic (m) cSCC who received cemiplimab (350 mg every three weeks) at the Center for Immuno-Oncology, University Hospital of Siena, Italy, either through an Expanded Access Program or routine clinical practice. Clinical outcome and treatment related adverse events (TRAEs) are reported. Results: Between December 2019 and December 2023, 27 patients (24 male; median age 82 years [range 41–90]) diagnosed with lacSCC (*n* = 20 [74.0%]) or mcSCC (*n* = 7 [25.9%]) were treated with cemiplimab as first line therapy and were followed until June 2024. Head and neck were the primary tumor location for 88.8% of patients, followed by trunk (7.4%) and lower extremities (3.7%). All patients had comorbidities, including six patients (22.2%) with hematologic malignancies. With a median follow-up of 31 months (data cut-off June 2024), the ORR was 66.6% (complete response 22.2%) and the disease control rate (DCR) 77.7%. Median progression-free survival (mPFS) and overall survival (mOS) were not reached, while 2-year PFS and OS rates were 65.2% and 71%, respectively. Treatment was well-tolerated, with three (11.1%) patients experiencing grade ≥3 TRAEs, and three patients discontinuing treatment due to TRAEs. Conclusions: Our real-world experience confirms the high rate of durable objective responses, good tolerability and long treatment duration of cemiplimab in elderly and frail cSCC patients as well.

## Simple Summary

Cutaneous squamous cell carcinoma (cSCC) is a life-threatening disease when no longer amenable to curative surgery or radiotherapy. Results from clinical trials in patients with locally advanced or metastatic cSCC have led to the availability of the anti-PD-1 monoclonal antibody cemiplimab in the daily practice, dramatically changing the course of the disease. Thus, real-world experience is crucial for assessing the efficacy and safety of treatment also in unselected patients outside clinical trials. Here we report a real-world experience with cemiplimab in locally advanced or metastatic cSCC patients treated at the Center for Immuno-Oncology of the University Hospital of Siena, Italy. Our results are consistent with the high rate of durable objective responses, the benefits of prolonged treatment, and the good tolerability of cemiplimab previously observed in clinical trials in elderly patients with multiple comorbidities.

## 1. Introduction

Cutaneous squamous cell carcinoma (cSCC) is the second most common non-melanocytic skin cancer, with an increasing incidence predominantly among elderly patients [1,2]. cSCC is caused by the malignant proliferation of epidermal keratinocytes driven by germline, somatic and epigenetic alterations, environmental interactions and/or escape from immunosurveillance [3,4]. The most important risk factor associated with the development of cSCC is ultraviolet radiation (UVR) exposure [5,6]; other known risk factors include immunosuppression, certain drugs (e.g., BRAF inhibitors, immunosuppressive agents, thiazide diuretics), beta—human papilloma virus (ß—HPV), and smoking [5,7,8,9]. The majority of patients have limited disease that can be successfully treated with local therapies such as surgical excision, cryotherapy, curettage, topical fluorouracil or imiquimod, intralesional INFα and local radiotherapy [8,10,11,12]. It is estimated that across different study populations, healthcare settings and underlying risk factors, only 5% of cSCC are unresectable, locally advanced (lacSCC) or with local or distant metastasis (mSCC) requiring systemic therapy [13]. cSCC most commonly develops in chronically sun-exposed areas such as the head and neck; thus, invasive surgical resections significantly impact the quality of life [4]. Historically, advanced cSCC has been treated with poorly effective platinum-based chemotherapy regimens that may provide temporary benefit with partial responses in approximately 40% of patients; however, these agents are generally poorly tolerated by elderly and frail patients due to age-related organ functional decline and comorbidity [14,15].

Over the last decade, a better understanding of the immune biology of cancer has led to the identification of novel, more effective and better-tolerated therapeutic strategies. A major breakthrough was achieved with the evidence that, while the immune system can fight cancer, tumor cells are able to evade immune surveillance. This comprises the expression of immune checkpoint molecules, such as programmed death-ligand 1 (PD-L1) and CTLA-4. PD-1 is a cell surface receptor that acts as a T cell checkpoint, playing a pivotal role in the regulation of T cell exhaustion. The binding of PD-1 to its ligand, PD-L1, activates downstream signaling pathways and inhibits T cell activation. Furthermore, abnormally high PD-L1 expression on tumor cells and antigen-presenting cells within the tumor microenvironment facilitates the tumor immune escape [16]. Along this line, immunotherapy targeting PD-1/PD-L1 immune checkpoints has reshaped treatment paradigms across several cancers [17]. Cutaneous SCC is characterized by a high tumor mutational burden (TMB) [18] and upregulation of immune checkpoint molecules (e.g., PD-1/PD-L1) which promote peripheral T cell exhaustion [19]. Furthermore, the strong association between cSCC and immunosuppression may indicate a role for the natural immune surveillance in controlling the disease. This notion strongly supports the use of therapeutic approaches that enhance the antitumor immune response, such as Immune Checkpoint Inhibitors (ICIs) [13]. In this context, the introduction of cemiplimab, a human monoclonal IgG4 anti-PD-1 antibody, has dramatically changed the course of cSCC. Based on the phase II EMPOWER-CSCC 1 study [20] showing an objective response rate (ORR) of 47.2% (95% CI 39.9–54.4) and a median duration of response (mDOR) of 41.3 months (95% CI 38.8–46.3), cemiplimab received the approval by the Food and Drug Administration (FDA) and the European Medicine Agency (EMA) as a first-line treatment for unresectable, locally advanced or metastatic cSCC. However, real-world experience remains crucial, since patients treated in clinical practice are typically elderly and with comorbidities, and with an overall poor performance status. Published real-world data have included patients with a median age ranging from 77 to 81 years, and with several comorbidities, secondary malignancies, organ transplants, or receiving immunosuppressive treatments [21,22,23,24,25]. However, the optimal duration of treatment and whether to stop cemiplimab after a response has been achieved are still open questions that could affect clinical practice. Here, we present real-world experience of lacSCC and mcSCC patients treated with cemiplimab at the Center for Immuno-Oncology of the University Hospital of Siena, Italy.

## 2. Materials and Methods

### 2.1. Patients

Eligible patients were adults (>18 years old) with advanced cSCC who were treated with cemiplimab intravenously (at least 1 cycle) every three weeks either within an Expanded Access Program (EAP) or per standard of care (SOC) at the Center for Immuno-Oncology of the University Hospital of Siena, Italy. Patients were classified as lacSCC or mSCC according to the European consensus-based interdisciplinary guidelines [26]. Patients with an ECOG performance status of 0–2, comorbidities, or with a previous second malignancy were eligible. All patients signed a written informed consent prior to receiving cemiplimab as standard treatment or as part of the EAP approved by the Ethics Committee of the University Hospital of Siena.

### 2.2. Study Design

This is a retrospective, real-word analysis of consecutive cSCC patients who started treatment with cemiplimab between December 2019 and December 2023 at the Center for Immuno-Oncology of the University Hospital of Siena, Italy and were followed until 1 June 2024. Cemiplimab was administered intravenously at the standard dose of 350 mg every three weeks until progression of disease (PD), unacceptable toxicity or the physician/patient’s decision. The response was assessed using serial photographic evaluations of superficial cutaneous lesions, and/or with computed tomography (CT), magnetic resonance imaging (MRI), or positron emission tomography (PET/TC) scans. Progression-free survival (PFS) and overall survival (OS) were also reported. All reported adverse events (AEs) were graded according to the Common terminology Criteria for Adverse Events (CTCAE) version 5.0.

### 2.3. Statistical Analysis

All data collected were entered into an anonymized database and were analyzed as a single cohort. Descriptive statistics were used to analyze demographic data and disease characteristics. The median follow-up was estimated using the Kaplan–Meier reverse method. The efficacy of cemiplimab was assessed according to the Response Evaluation Criteria in Solid Tumors (RECIST), version 1.1. The objective response rate (ORR) was defined as the proportion of patients achieving a partial response (PR) or complete response (CR); disease control rate (DCR) was defined as the proportion of patients achieving either a PR, CR or stable disease (SD). PFS was defined as the time from the first administration of cemiplimab until progression of disease (PD) or death from any cause. OS was defined as time from the first administration of cemiplimab until death from any cause; patients lost to follow-up or continuing treatment at other hospitals were censored at the time of the last tumor assessment or the last date known to be alive, respectively. Survival analysis was performed using the Kaplan–Meier method and the 2-year rate reported. The association between patient disease characteristics and OS was investigated through Cox regression analysis, and Hazard Ratios with their 95% confidence intervals were reported. Adverse events graded according to the CTCAE version 5.0 were described.

## 3. Results

### 3.1. Patients and Treatment

A total of 27 patients were included and analyzed in this single-center experience. The majority of patients were male (24 patients, 88.8%), median age was 82 years (range 41–90), and 18 (66.6%) patients were ≥75 years old. Twenty patients (74.0%) had lacSCC, and seven patients (25.9%) mcSCC. Six patients had secondary hematological malignances (chronic lymphocytic leukemia, myelofibrosis, marginal cell lymphoma, JAK2-related myeloproliferative disease).

Baseline characteristics are reported in Table 1. None of the patients had received prior systemic therapies for cSCC, while three patients (11.1%) had received prior adjuvant radiotherapy. At the time of the analysis, patients had received a median number of 24 cycles (range 2–63) of cemiplimab. The median duration of treatment was 16.5 months (range 1–47), seven patients (25.9%) received cemiplimab for more than 2 years and nine were still on treatment. For the 18 patients (66%) who had discontinued treatment, the primary reasons for discontinuation were disease progression (*n* = 5; 18.5%), the physician’s decision after achieving a CR (*n* = 3; 11.1%), immune-related adverse event (*n* = 3; 11.1%), the patient’s decision (*n* = 3; 11.1%), worsening of underlying comorbidities (*n* = 3; 11.1%), and continuation of treatment at another hospital (*n* = 1; 3.7%).

### 3.2. Treatment Efficacy

**Figure 1:**
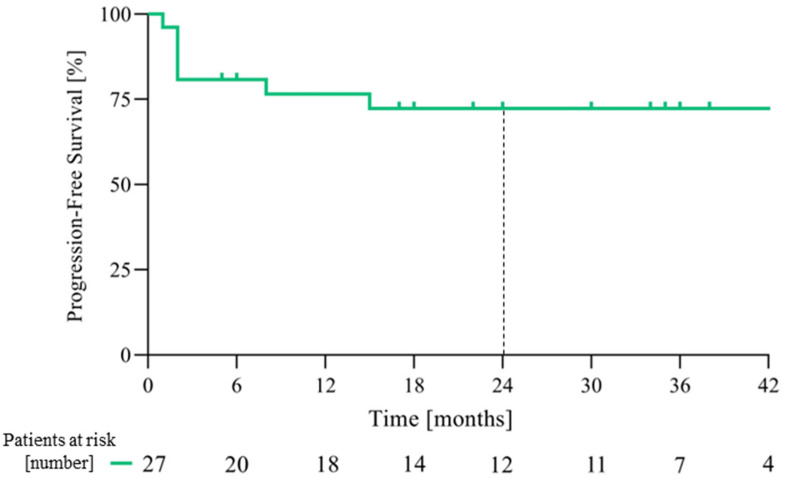
Kaplan–Meier estimates of progression-free survival.

**Figure 2:**
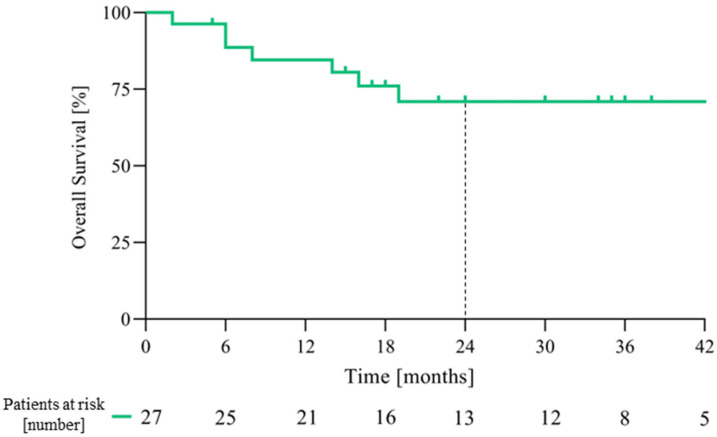
Kaplan–Meier estimates of overall survival.

**Figure 3:**
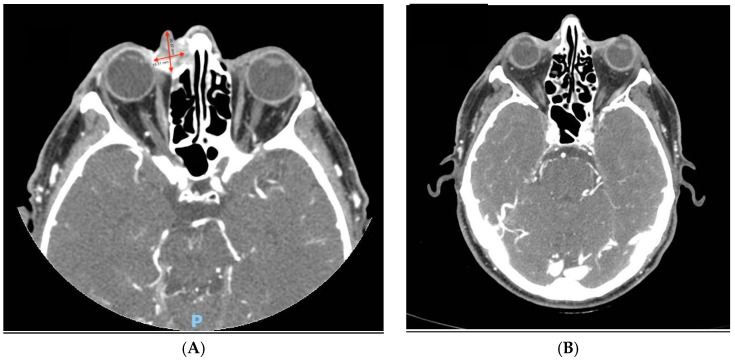
A 74-year-old male experienced impaired vision and ocular pain two weeks after the surgical excision of a cSCC at the medial canthus of his right eye. A head CT scan revealed a disease recurrence with evidence of a heteroplastic mass (12 × 20 × 21 mm, red arrows) with local invasion and bone involvement (**A**). To avoid a wide excision with exenteration of the orbit, treatment with cemiplimab was started. Following the first cycle, significant tumor regression was observed, resulting in a CR after 4 months (**B**). At the time of the analysis, the CR had been ongoing for more than one year and patient had regained complete vision.

**Figure 4:**
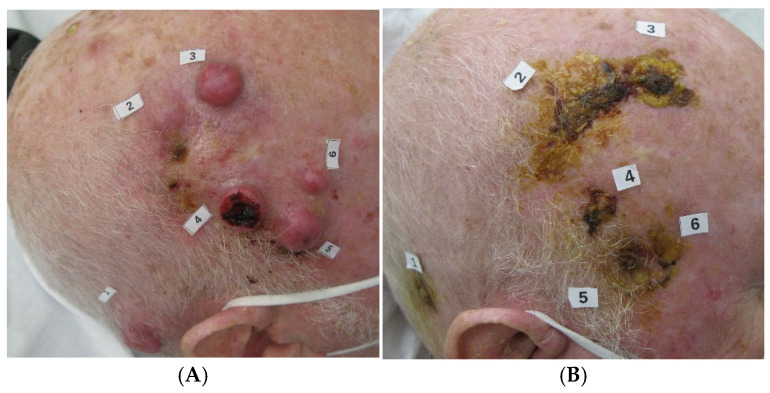

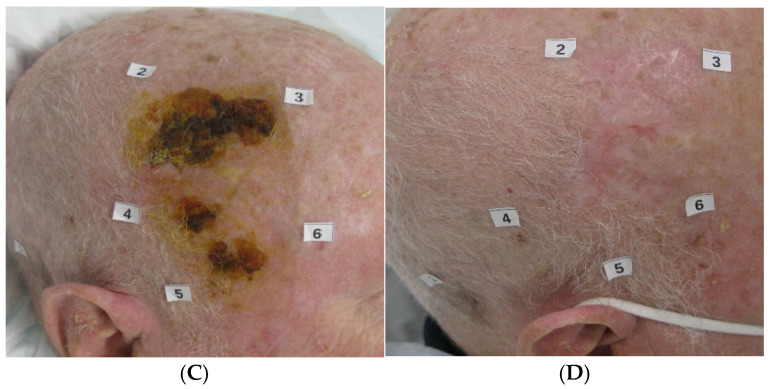
An 82-year-old man with severe cardiological comorbidities and multiple painful lesions (target lesions numbers 1–6) of the scalp due to recurrent cSCC, not amenable to either surgery or radiotherapy, (**A**) was treated with cemiplimab. Following the first 2 cycles of treatment, a PR was reported (**B**) with continued improvement after 5 months (**C**). The patient achieved a CR after 9 months of therapy (**D**). At the time of the analysis, the patient had discontinued cemiplimab for over 2 years without experiencing a relapse.

At the data cut-off date of 1 June 2024 and with a median follow-up of 31 months (range 1–49, IQR 17–38), the median PFS (Figure 1) and OS (Figure 2) were not reached. The PFS and OS rates at 2 years were 65.2% (95% CI: 46.8–83.6) and 71.0% (95% CI: 52.6–89.4), respectively (Table 2). An objective response (OR) was observed in 18 patients (66.6%, 95% CI: 46.0–83.5), with 6 patients (22.2%) achieving a CR (Table 2); the disease control rate (DCR) was 77.7% (95% CI: 59.9–89.6). The median time to the first evidence of response was 2 months (range 2–9) and the median time to CR was 13.5 months (range 7–32). The median duration of response (mDOR) was 31 months (range 4–48), with 16 (59.2%) patients experiencing a response lasting more than one year. Two paradigmatic clinical cases among patients who achieved an objective response are also reported (Figure 3 and Figure 4).

The twelve (44.4%) patients who achieved disease control and discontinued cemiplimab maintained the response for a median of 10 months (range 0–28); the duration of response was longer in patients who had achieved a CR (13.8 months). None of the five patients who discontinued cemiplimab after achieving a CR had experienced disease progression at the time of the analysis.

Among the six patients who experienced a PD, two patients had local progression (scalp and lower extremity) and received concomitant radiotherapy during treatment with cemiplimab. Among the latter, one discontinued cemiplimab subsequently due to worsening of the underlying myeloproliferative disease, and the other continued cemiplimab at another hospital and was followed for survival.

No statistically significant association between OS and the following patient/disease characteristics was reported in our cohort: age > 75 years (HR 0.93; 95% CI: 0.22–3.90), corticosteroid use (HR 0.48; 95% CI: 0.11–2.04), lacSCC diagnosis (HR 0.97; 95% CI: 0.18–5.21), and history of lymphoproliferative malignancies (HR 1.04; CI 0.20–5.23).

### 3.3. Safety

Overall, 13 patients (48.1%) experienced treatment-related adverse events (TRAEs) of any grade (G) and 3 patients (11.1%) had G ≥ 3 TRAEs (see Table 3); no G5 adverse events (AEs) were reported. The most common TRAEs of any grade were cutaneous rash (18.5%), pruritus (7.4%), and diarrhea (7.4%). The G ≥ 3 AEs were myositis, haemolytic anemia, hyperlipasemia and hypertransaminasemia; one patient experienced two concomitant G4 TRAEs of myositis and hypertransaminasemia. Three patients discontinued cemiplimab due to TRAEs (G2 pneumonia *n* = 1, G4 hypertransaminasemia *n* = 1, and *n* = 1 concomitant G4 myositis and haemolytic anemia). Eleven out of the thirteen patients (40.7%) who experienced TRAEs were treated with corticosteroids (1 mg/kg prednisone or equivalent). Among the six patients with concomitant hematological malignances, only two experienced G ≥ 2 TRAEs (i.e., hyperamylasemia, pneumonitis and hypercreatinemia).

## 4. Discussion

Advanced cSCC is a life-threatening disease when no longer amenable to curative surgery or radiotherapy. It predominantly affects elderly people with multiple comorbidities, causing painful and disfiguring lesions on the head and neck that severely affect quality of life (QoL). Thus, advanced cSCC represents a clinical challenge requiring a multidisciplinary approach to achieve the best outcome and preserve patients’ QoL. Immune Checkpoint Inhibitor (ICI)-based immunotherapy has changed the course of cSCC delivering durable objective responses and improved survival rates [2,20,27,28]. While results from pivotal trials are essential in providing patients with new treatment options, real-world experience is crucial in assessing the efficacy and safety of novel therapeutic strategies in a broader, unselected patient population, with the aim of optimizing treatment approaches. In this regard, our real-world study reports the efficacy and safety of cemiplimab in patients with advanced cSCC. With an ORR of 66.6% (22.2% CR) the results of our case series are consistent with those from clinical trials. Furthermore, with a median follow-up of 31 months, the mPFS and mOS had not been reached, and 65.2% of patients were alive and without progression after 24 months. Thus, the outcomes of cSCC patients treated in our cohort were comparable to those reported in registrational trials and in real-world studies [20,23,24,29]. Notably, our patient population included mainly previously untreated patients with locally advanced disease (74%) who achieved objective responses to cemiplimab as first-line therapy, which could explain the high objective response rate. These findings are consistent with those of Rischin et al. [30], who demonstrated the enhanced efficacy of cemiplimab in the treatment of cSCC when used as first-line therapy. This supports the early use of cemiplimab in patients with advanced cSCC.

Many real-world studies have investigated the role of clinical and biohumoral factors in predicting the response to cemiplimab. Baggi et al. [24] reported an association between response and the location of cSCC in the head and neck areas (*p* = 0.007), as well as normal hemoglobin values (*p* = 0.034). In contrast, a worse outcome was associated with performance status ≥1 (*p* = 0.012), chronic corticosteroids therapy (*p* = 0.038), previous radiation therapy to lymph nodes (*p* = 0.052) and previous chemotherapy (*p* = 0.0020). In addition, a recently published real-world prospective study conducted in the Netherlands [29] reported that auto-immune diseases, the use of corticosteroids or other immunosuppressive drugs did not significantly affect the clinical outcome. Instead, the presence of hematological malignancies was significantly associated with lack of both objective and physician-assessed clinical benefit (*p* = 0.022 and *p* = 0.007) [29]. In this scenario, our case series has shown no statistically significant association between OS and age, stage of disease, concurrent hematological malignancies, primary site of cSCC or the use of corticosteroids during treatment with cemiplimab. This may be likely due to the small sample size rather than true absence of association.

Furthermore, similarly to the evidence reported in published retrospective studies [25,31], the median time to first evidence of response in our cohort was 2 months (range 2–9), suggesting the early onset of the clinical benefit. Additionally, the median time to CR we have reported was 13.5 months (range 7–32), implying that responses deepen over time and supporting a prolonged treatment duration. Along this line, a key and still open question in clinical practice is whether cemiplimab can be discontinued earlier once the best response has been achieved. Published real-life studies have reported treatment durations with cemiplimab ranging from less than one month to 24 months [23,24,31]. Interestingly, the median treatment duration for patients who achieved a response in a cohort study in the Netherlands was 12.3 months (range 2.1–24.1) [31]. Additionally, a real-world evidence study of 105 patients with advanced cSCC who were treated with cemiplimab in the United Kingdom [32] showed an increase in best response rates with prolonged duration of treatment rising from 38% (95% CI, 29–47%) to 45% (95% CI 35–54%) at 6 and 36 months, respectively, after initiation of cemiplimab treatment. Similarly, CR rates increased from 5% to 12% between 6 and 12 months of treatment. Finally, Boutros et al. conducted a retrospective analysis of the occurrence of progression after discontinuing cemiplimab treatment in 48 patients with advanced cSCC. Of the 19 patients who discontinued cemiplimab, at a median follow up of 9.2 months, 16 patients did not experience progression after achieving SD, PR or CR [33]. In our case series, five patients discontinued cemiplimab after achieving a CR, which they maintained for a median of 13.8 months after their last dose of cemiplimab. At the time of this analysis, none of these patients had experienced disease progression. Interestingly, none of the seven patients (25.9%) who received cemiplimab for more than 2 years experienced disease progression. Despite the limitations of the small sample size, these findings suggest that cemiplimab therapy could be continued until either clinical benefit or unacceptable toxicity occurs, despite the common practice of stopping cemiplimab therapy after 24 months.

The safety profile of cemiplimab in our cohort was consistent with that reported in the literature and was overall acceptable for elderly patients with comorbidities [23,24,25,29,31]. The most frequent TRAEs were rash, diarrhea and pruritus, and were mainly G1 or 2. Three patients (11.1%) experienced G ≥ 3 TRAEs. Three patients (11.1%) discontinued cemiplimab due to a G ≥ 2 treatment-related TRAEs (i.e., autoimmune pneumonia, myositis, hemolytic anemia). This discontinuation rate due to TRAEs is comparable with available real-world data which reports a rate ranging from 7% to 18.5% [23]. Furthermore, cemiplimab has a manageable safety profile and it is well-tolerated by patients with several comorbidities.

Although our experience contributes to the evidence of long-term effectiveness of first-line cemiplimab therapy in elderly patients with comorbidities and provides insight on the optimal duration of treatment, it has limitations. The retrospective nature of the study, the small number of patients, mainly lacSCC (74%), enrolled at a single center, and the relatively short follow-up after discontinuation of cemiplimab limit the statistical power and the ability to reach definitive conclusions. Future larger multicenter studies with a longer follow-up are needed to validate our findings and to address the question of the optimal duration of treatment with cemiplimab.

## 5. Conclusions

Our real-word results showed the long-term benefits of cemiplimab for elderly patients with advanced cSCC. Patients who received cemiplimab treatment for more than two years continued to respond to the treatment, suggesting that cemiplimab should be administered until objective response is achieved or unacceptable toxicity occurs. Furthermore, cemiplimab has a manageable safety profile and it is well-tolerated by patients with several comorbidities.

## Figures and Tables

**Table 1:** Patient demographic and baseline disease characteristics.

Parameter	Number (%) (*n* = 27)
Age, median (range),	82 (41–90)
<75	9 (33.3%)
≥75	18 (66.6%)
Sex, *n* (%)	
Female	3 (11.1%)
Male	24 (88.8%)
ECOG PS, *n* (%)	
0	6 (22.2%)
1	20 (70.0%)
2	1 (3.7%)
Comorbidities *n* (%)	27 (100%)
Second malignancy	11 (40.74%)
Hematological	6 (22.2%)
Cutaneous	3 (11.1%)
Neuro-endocrine	1 (3.7%)
Bone	1 (3.7%)
Cardiovascular	21 (77.7%)
Endocrine	9 (33.3%)
Pulmonary	5 (18.5%)
Renal/urological	7 (25.9%)
Gastrointestinal	9 (33.3%)
Primary cSCC site	
Head/neck	24 (88.8%)
Trunk	2 (7.4%)
Extremity	1 (3.7%)
Histological differentiation	
Moderately	19 (70.37%)
Poorly	2 (7.4%)
Unknown	6 (22.2%)
Stage	
Locally advanced	20 (74.0%)
Metastatic	7 (25.9%)
Prior SLNB	
Yes	4 (14.8%)
Positive	2
Negative	1
Unknown	1
No	23 (85.1%)
Prior systemic therapy	0 (0%)
Prior radiotherapy	3 (11.1%)

cSCC, Cutaneous squamous cell carcinoma; SLNB, sentinel lymph node biopsy.

**Table 2:** Efficacy outcome.

	All Patients *N* = 27
ORR, *n* (%; 95% CI)	18 (66.6%; 46.0–83.5)
BOR, *n* (%)	
CR	6 (22.2%)
PR	12 (44.4%)
SD	3 (11.1%)
PD	6 (22.2%)
DCR, *n* (%; 95% CI)	21 (77.7%; 59.9–89.6)
Time to response, median, (range) months *	2 (2–9)
DOR, median, (range) months	31 (4–48)
≥12 mo (%)	16 (59.2%)
PFS, median, months	NR
24-month PFS rate	65.2% (95% CI 46.8–83.6)
OS, median, months	NR
24-month OS rate	71% (95% CI 52.6–89.4)

* Time to first evidence of tumor response assessed either by radiologic or clinical evidence. ORR, objective response rate; BOR, best overall response; CR, complete response; PR, partial response; SD, stable disease; PD, progressive disease; DCR, disease control rate; DOR, duration of response; PFS, progression-free survival; OS, overall survival; NR, not reached.

**Table 3:** Treatment-related adverse events.

Event	Any Grade N (%)	Grade ≥ 3 N (%)
Any treatment-related adverse event	13 (48.1%)	4 (14.8%)
Rash	5 (18.5%)	0
Pruritus	2 (7.4%)	0
Diarrhea	2 (7.4%)	0
Mucosytis	1 (3.7%)	0
Hypophysitis	1 (3.7%)	0
Hypothyroidism	1 (3.7%)	0
Arthralgia	1 (3.7%)	0
Pneumonitis	1 (3.7%)	0
Autoimmune hemolytic anemia	1 (3.7%)	1 (3.7%)
Myositis	1 (3.7%)	1 (3.7%)
Hypertransaminasemia	1 (3.7%)	1 (3.7%)
Hyperlipasemia	1 (3.7%)	1 (3.7%)

## Data Availability

The original contribution and data presented in this study are included in the article. Further data can be asked directly to the corresponding author.

## Abbreviations

The following abbreviations are used in this manuscript:

AE	Adverse event
ALT	Alanine aminotransferase
AST	Aspartate aminotransferase
CI	Confidence interval
CR	Complete response
CT	Computed tomography
CTCAE	Common terminology criteria for adverse events
DCR	Disease control rate
EAP	Expanded access protocol
ECOG PS	Eastern Cooperative Oncology Group performance status
EMA	European Medicina Agency
FDA	Food and Drug Administration
ICI	Immune checkpoint inhibitors
MRI	Magnetic resonance
NR	Not reached
ORR	Objective response rate
OS	Overall survival
QoL	Quality of life
SCC	Squamous cell carcinoma
SD	Stable disease
cSCC	Cutaneous squamous cell carcinoma
lacSCC	Locally advanced cutaneous squamous cell carcinoma
mSCC	Metastatic cutaneous squamous cell carcinoma
mDOR	Median duration of response
SOC	Standard of care
PET/TC	Positron emission tomography
PD	Progression disease
PFS	Progression-free survival
PR	Partial response
TMB	Tumor mutational burden
TRAEs	Treatment related adverse events
UVR	Ultraviolet radiation
